# E3 ubiquitin ligase RNF126 affects bladder cancer progression through regulation of PTEN stability

**DOI:** 10.1038/s41419-021-03521-1

**Published:** 2021-03-04

**Authors:** Huimin Xu, Lingao Ju, Yaoyi Xiong, Mengxue Yu, Fenfang Zhou, Kaiyu Qian, Gang Wang, Yu Xiao, Xinghuan Wang

**Affiliations:** 1 Department of Urology, Zhongnan Hospital of Wuhan University, Wuhan, China; 2 Department of Biological Repositories, Zhongnan Hospital of Wuhan University, Wuhan, China; 3 Human Genetic Resource Preservation Center of Hubei Province, Wuhan, China; 4 Wuhan Research Center for Infectious Diseases and Cancer, Chinese Academy of Medical Sciences, Wuhan, China; 5 Laboratory of Precision Medicine, Zhongnan Hospital of Wuhan University, Wuhan, China; 6 Medical Research Institute, Wuhan University, Wuhan, China

**Keywords:** Bladder cancer, Diagnostic markers

## Abstract

E3 ubiquitin ligase RNF126 (ring finger protein 126) is highly expressed in various cancers and strongly associated with tumorigenesis. However, its specific function in bladder cancer (BCa) is still debatable. Here, we found that *RNF126* was significantly upregulated in BCa tissue by TCGA database, and our studies indicated that downregulation of *RNF126* significantly inhibited cell proliferation and metastasis through the EGFR/PI3K/AKT signaling pathway in BCa cells. Furthermore, we identified PTEN, an inhibitor of the PI3K/AKT signaling pathway, as a novel substrate for RNF126. By co-immunoprecipitation assays, we proved that RNF126 directly interacts with PTEN. Predominantly, PTEN binds to the C-terminal containing the RING domain of RNF126. The in vivo ubiquitination assay showed that RNF126 specifically regulates PTEN stability through poly-ubiquitination. Furthermore, *PTEN* knockdown restored cell proliferation, metastasis, and tumor formation of BCa cells inhibited by *RNF126* silencing in vitro and in vivo. In conclusion, these results identified RNF126 as an oncogene that functions through ubiquitination and degradation of PTEN in BCa.

## Introduction

Bladder cancer (BCa) is the 10th most common cancer worldwide, with an estimated 81,400 new cases and 17,980 deaths in the United States alone in 2020^1,2^. Approximately 75% of non-muscle-invasion bladder cancer (NMIBC) will relapse or progress to muscle-invasive bladder cancer (MIBC), which possesses a feature of rapid growth and metastasis^3,4^. MIBC is a life-threatening disease, the five-year overall survival rate is around 60% because of distant metastasis, even after receiving chemotherapy and surgery^5^. Therefore, it is critical to identify new molecular targets that specifically regulate the progression of BCa for improving the treatment of invasive BCa patients.

RNF126 is a RING domain-containing protein that plays an important role in different physiological processes dependent or independent of the E3 ligase activity. A bunch of substrates of E3 ligase RNF126 have been identified, including G0/G1 switch gene 2 (G0S2)^6^, frataxin (FXN)^7,8^, epidermal growth factor receptor (EGFR)^9^, pyruvate dehydrogenase kinases (PDKs)^10^, insulin-like growth factor II receptor (IGF-IIR)^11^, mechanistic target of rapamycin (mTOR)^12^, CDK inhibitor CDKN1A/p21^13^ and Bag6 complex^14^. As a transcription factor, RNF126 promotes DNA homologous recombination (HR) through interacting with E2F1 and promoting the transcription of *BRCA1*^15^. Recently, RNF126 was reported to interact with two key E3 ubiquitin ligases RNF8 and RNF168 related to the HR^16^. In addition, Ishida et al. proved that RNF126 promoted the process of non-homologous end joining (NHEJ) mediated DNA repair by polyubiquitinating Ku80^17^.

PTEN (phosphatase and tension homolog) is known as a tumor suppressor gene, often deleted or highly mutated in diversified cancers^18,19^. Germline mutations or deletions of PTEN are associated with the tumor predisposition syndromes, such as Bannayan–Zonana syndrome, Cowden syndrome, and Lhermitte-Duclos disease^20–23^. Functionally, the lipid phosphatase PTEN was characterized as a non-redundant negative regulator of the PI3K/AKT pathway regulating multiple cellular processes, including cell proliferation, survival, metastasis and apoptosis^24,25^. PTEN dephosphorylates the secondary messenger PIP3 to PIP2, thereby depleting cellular PIP3, critical for AKT activation. In addition to PTEN germline mutation in various cancers, diversified post-translational modifications (PTM) of PTEN have been broadly recognized, such as acetylation, phosphorylation, oxidation and ubiquitination/deubiquitination. These PTMs regulated the activity and stability of PTEN to suppress the PI3K/AKT signaling pathway^26–28^. Additionally, ubiquitination plays one of the most significant roles in regulating PTEN degradation and compartmentalization^29^. Several E3 ligases and deubiquitinases of PTEN were discovered, including Nedd4-1^30^, X-linked inhibitor of apoptosis (XIAP)^31^ and WW domain-containing E3 ubiquitin protein ligase 2 (WWP2)^32^, carboxyl terminus of HSC70-interacting protein (CHIP)^33^, ubiquitin specific peptidase 7 (USP7)^34^ and ubiquitin specific peptidase 13 (USP13)^35^. It seemed to show that PTEN stability is essential in cancer progression. However, the regulation of PTEN is complex and the mechanisms to regulate the steady levels remain poorly understood.

Although RNF126 has been reported to function as an oncogene in several cancers, including breast cancer^15^, prostate cancer^13^, gastric cancer^36^, leukemia^12^, and tongue cancer^37^, the role of RNF126 in BCa remains unclear.

Here, we revealed a novel mechanism for RNF126 in promoting BCa and identified PTEN, a critical negative regulatory factor in the PI3K/AKT pathway, as a new RNF126 substrate. We have described that RNF126 interacts with PTEN and mediates its ubiquitination and proteasomal degradation in BCa cells. These may function to fine-tune the PI3K/AKT pathway and contribute to carcinogenesis.

## Results

### RNF126 is highly expressed in BCa tissues and cell lines

**Fig. 1:**
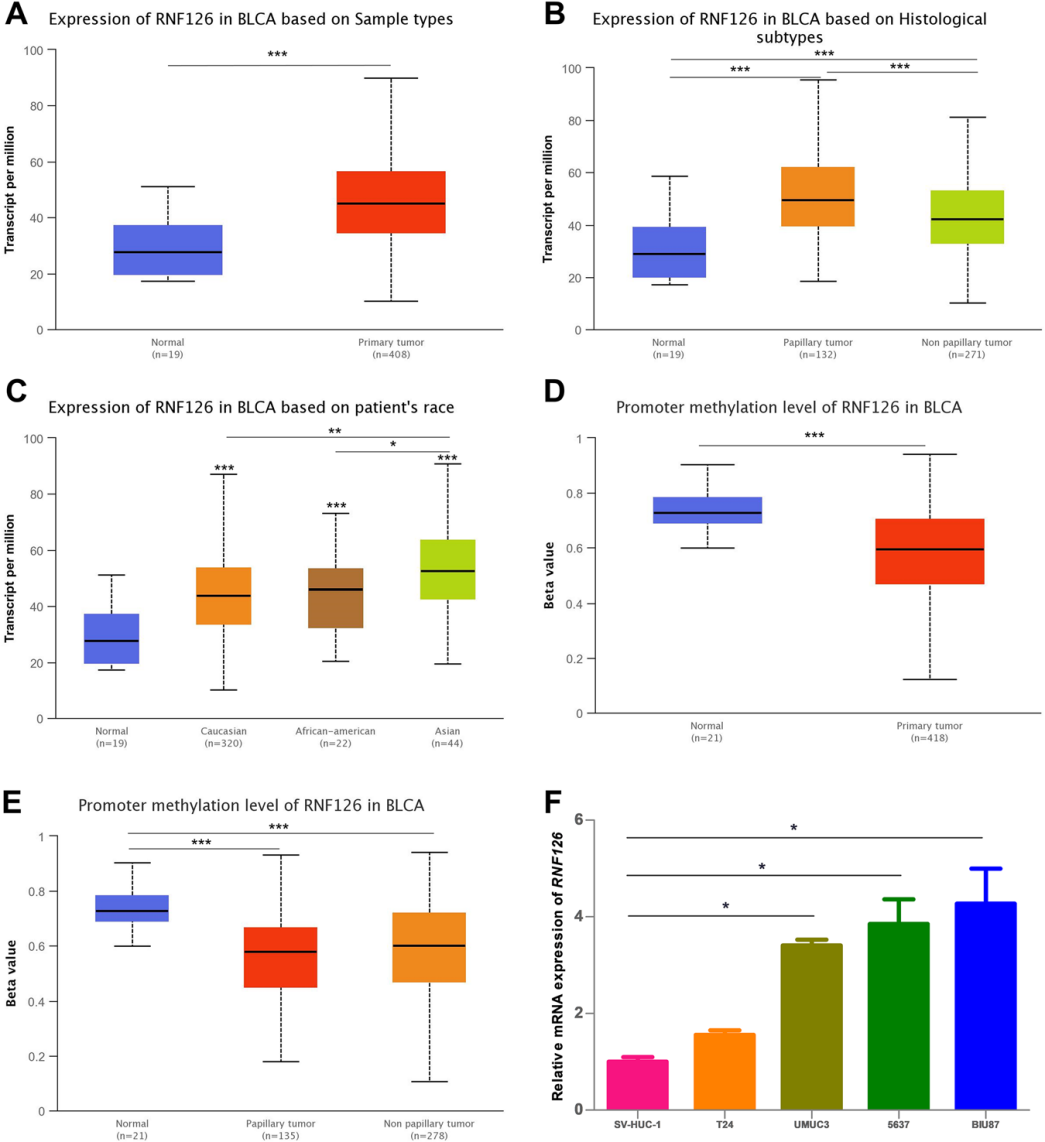
Expression of RNF126 in BCa of TCGA samples and different cell lines. **A** *RNF126* expression in the normal and primary tumor of the bladder. **B** *RNF126* expression is based on different histological subtypes: papillary tumor and non-papillary tumor. **C** Expression of *RNF126* in BCa based on the patient’s race. **D** Promoter methylation level of *RNF126* in BCa. **E** *RNF126* promoter methylation profile is based on histological subtypes. **F** The qRT-PCR analysis confirmed the transcription level of *RNF126* in normal bladder epithelial cells SV-HUC-1 and various BCa cell lines (T24, UMUC3, 5637, BIU78). ***p < 0.001, **p < 0.01, *p < 0.05.

To identify and characterize the diagnostic markers of BCa, we examined the TCGA database and found an interesting candidate gene, *RNF126*, which was highly expressed in BCa. Compared with the normal human bladder tissue, the transcription level of *RNF126* in primary BCa tissue is significantly increased (Fig. 1A). Furthermore, the transcription level of *RNF126* in papillary carcinoma is higher than that in non-papillary carcinoma (Fig. 1B). Among different races with BCa, Asians have higher levels of *RNF126* than Caucasians and African-Americans (Fig. 1C). In addition, DNA methylation catalyzed by DNA methyltransferase (DNMTs), one of the fundamental epigenetic mechanisms that control cell proliferation, apoptosis, cell cycle and differentiation in cancers^38,39^. While the expression of *RNF126* is upregulated in BCa, and the promoter DNA methylation level of *RNF126* is downregulated (Fig. 1D, E). Other data of *RNF126* in TCGA were shown in Supplementary Fig. S1.

To further verify the differential expression of *RNF126* in BCa, the qRT-PCR analysis was performed to compare the expression of *RNF126* in human bladder epithelial cells SV-HUC-1 and various BCa cell lines (T24, UMUC3, 5637, BIU78). Finally, *RNF126* was found to be overexpressed in BCa cell lines (Fig. 1F). These results indicate a strong association between RNF126 expression and BCa progression, supporting this protein’s utility as a useful diagnostic marker for BCa.

### Depletion of RNF126 suppressed cell proliferation, migration, and cell cycle in BCa

**Fig. 2:**
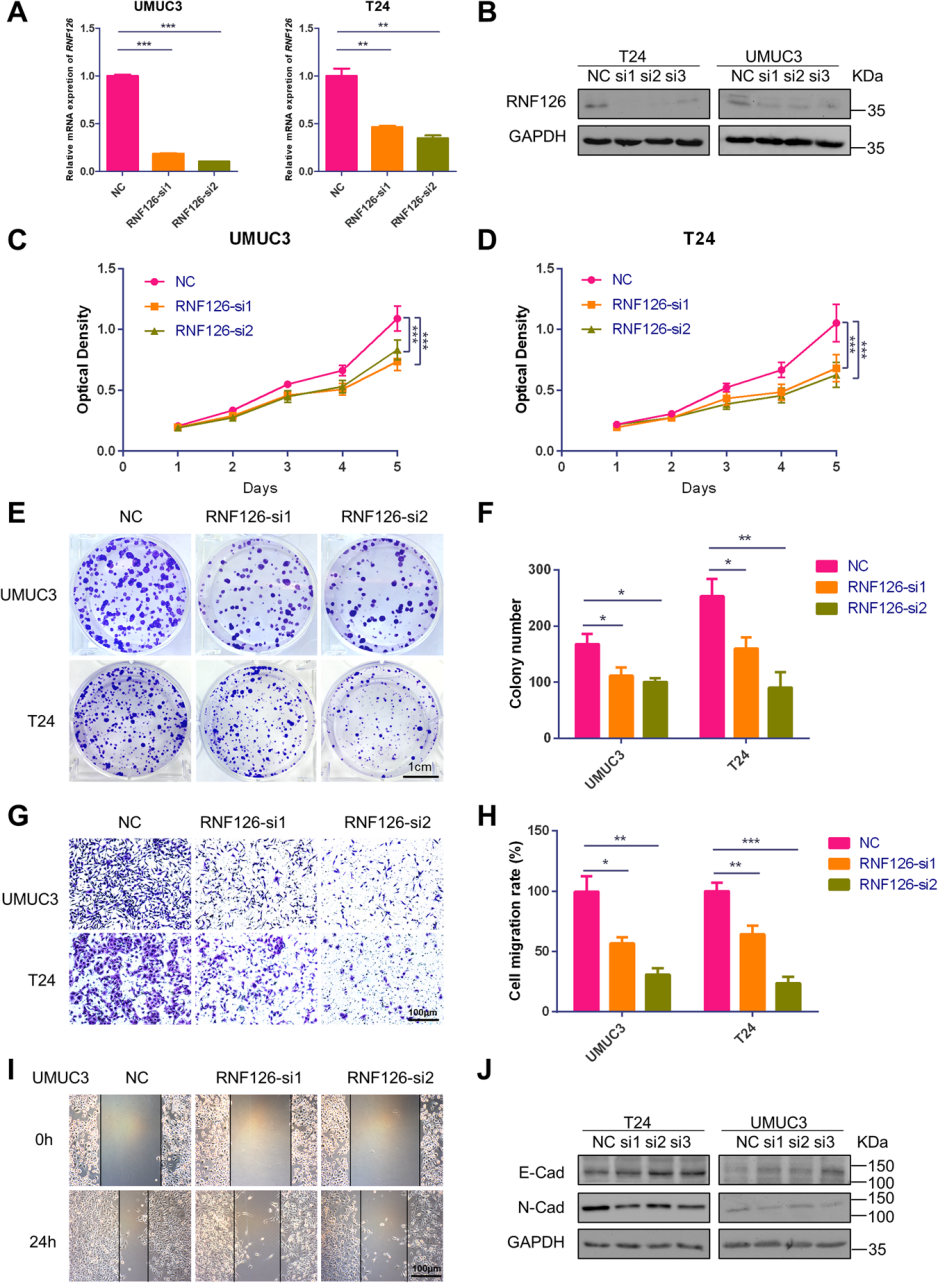
Depletion of RNF126 inhibited BCa cell proliferation, migration, and viability. **A** The qRT-PCR verified the efficiency of knockdown *RNF126* by siRNA (RNF126-si1 and RNF126-si2) in two BCa cells T24 and UMUC3. **B** The western blotting verified the expression of RNF126 downregulation in T24 and UMUC3 cells after transfection with the *RNF126* siRNA. The internal control was GAPDH abundance. **C, D** The MTT assay evaluated the T24 and UMUC3 cells growth and viability from day 1 until day 5 after transfection with the *RNF126*-si1 and si2 (orange line and green line). **E** The colony formation assay showed the effect of *RNF126* knockdown on the cell survival of UMUC3 and T24 cells after the transfection. Scale bar = 1 cm. **F** Colony numbers were counted and plotted as indicated. **G** The transwell assay evaluated cell migration of the *RNF126*-si1, si2 and NC treated BCa cells. Scale bar = 100 µm. **H** The number of cell migration was counted and statistically analyzed. **I** The wound healing assay evaluated the migration of *siRNF126*-treated BCa cells after scratching for 24 h. Scale bar = 100 µm. **J** The western blotting revealed the downregulation of protein abundance of EMT markers (E-Cad, N-Cad) in the BCa by reduction of RNF126. GAPDH severed as control. ***p < 0.001, **p < 0.01, *p < 0.05.

**Fig. 3:**
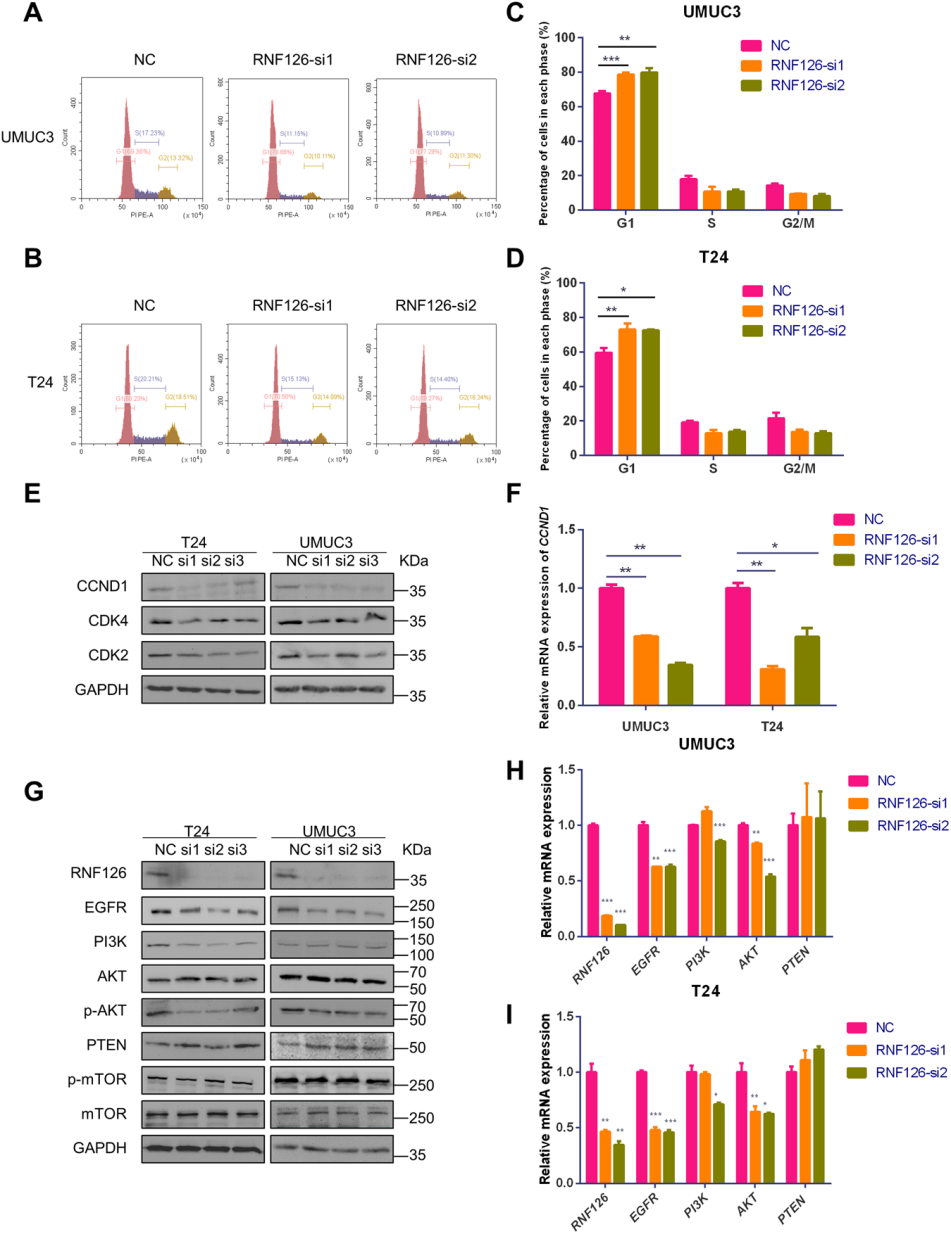
RNF126 deficiency affects the cell cycle and EGFR/PI3K/AKT signaling pathway in BCa cells. **A**, **B** The flow cytometry analysis demonstrated the percentage (%) of cells in different phases of cell cycle. The T24 and UMUC3 cells were treated with *RNF126*-si1 and si2 for two days. **C**, **D** The percentage (%) of cells in each phase was statistically analyzed from three independent experiments. **E** The western blotting revealed downregulation of protein associated with the cell cycle (CCND1, CDK2 and CDK4) in the BCa cells after silencing *RNF126*. The internal control was GAPDH. **F** The relative mRNA level of *CCND1* in different cell types, treatment of siRNA. **H** The western blotting showed the protein level of EGFR, PI3K, total and phosphorylated AKT, mTOR, and PTEN in BCa cells treated with *RNF126*-si1and si2. The internal control was GAPDH. **I** The relative mRNA level of *EGFR*, *PI3K*, *AKT*, *PTEN* in different cell types, and siRNA treatment. ***p < 0.001, **p < 0.01, *p < 0.05.

To study the functions of RNF126 on BCa, three independent siRNAs specific for *RNF126* were evaluated by western blotting (Figs. 2B, J, 3E, and G) and *RNF126* si1 and si2 were chosen for later experiments. Moreover, we constructed the RNF126 overexpression plasmid with the FLAG-tag to upregulate the expression of RNF126 (Supplementary Fig. S2A). In two BCa cell lines T24 and UMUC3, the qRT-PCR analysis confirmed the efficiency of mRNA level knockdown (Fig. 2A) and western blot analysis confirmed the same results for protein levels (Fig. 2B). Subsequently, MTT assay showed that the depletion of *RNF126* decreased the proliferation ability of UMUC3 and T24 cells (Fig. 2C, D). The effect of *RNF126* knockdown in colony formation was consistent with MTT assay (Fig. 2E, F). Transwell migration assay and wound healing assay both showed that knocking down *RNF126* suppressed cell migration in BCa cells (Fig. 2G–I). As is known, epithelial-mesenchymal transition (EMT) was related to cancer invasion and metastasis^40,41^. Proteins involved in the EMT process, including N-cad and E-cad, were analyzed by western blot analysis. The study exhibited an up-regulation of E-cad and downregulation of N-cad after *RNF126* silencing (Fig. 2J). This result was consistent with inhibition in tumor phenotypes (Fig. 2G–I) by knocking down *RNF126*. Flow cytometry analysis was performed to observed the cell cycle, revealed that cells blocked in the G1 phase when *RNF126* silenced (Fig. 3A–D). It is well known that CCND1 functions as a regulator of cyclin-dependent kinases (CDKs) and its essential role is to promote cell proliferation^42^. We found that *RNF126* knockdown significantly reduced both mRNA and protein levels of CCND1, causing cell cycle arrest and inhibiting cell proliferation (Fig. 3E, F). Also, overexpression of RNF126 can partly promote cell proliferation and migration (Supplementary Fig. S2B–F). Altogether, these results indicated that the depletion of *RNF126* repressed cell proliferation and metastasis in the BCa cells.

### RNF126 depletion potentiates the chemotherapy sensitivity to cisplatin by inducing increased apoptosis in BCa cells

In view of the critical role of RNF126 in DNA damage repair^16^, we supposed that RNF126 might promote apoptosis and chemoresistance of BCa. Because apoptosis is one of the essential mechanisms responsible for inducing cancer cell death by cisplatin^43^, we subsequently studied the role of RNF126 in cisplatin-induced apoptosis. Then, we treated the cells with cisplatin, a chemotherapeutic agent, in the most appropriate drug concentration of 5 μM (Supplementary Fig. S3A). The results demonstrated that the downregulation of *RNF126* had little effect on the apoptosis of BCa cells. Although *RNF126*-si2 and DMF treatment increased apoptosis in UMUC3 cells, *RNF126*-si1 and DMF treatment almost did not affect apoptosis (Supplementary Fig. S3B–E). The results revealed that the apoptotic rates of *siRNF126* cells were significantly increased compared with the control group after cisplatin treatment (Supplementary Fig. S3B–E). Therefore, our results indicated that knockdown of *RNF126* could significantly increase the chemotherapy sensitivity of BCa cells.

### RNF126 affects EGFR/PI3K/AKT signaling pathway

The previous study demonstrated that RNF126 promotes tongue cancer progression through PI3K/AKT signaling pathway^37^. To further investigate the underlying mechanisms of RNF126 regulated tumorigenesis in BCa, we performed western blotting to analyze various carcinogenesis-related genes of PI3K/AKT signaling pathway. *RNF126* silencing resulted in a substantial reduction of EGFR and phosphorylated AKT, while PI3K was slightly reduced, but the protein level of PTEN is significantly increased in T24 and UMUC3 cells (Fig. 3G). To further study whether RNF126 changed the transcription level of these genes, we performed the qRT-PCR analysis in two BCa cell lines. Our results indicated that the mRNA expression of *EGFR* and *AKT* were downregulated. Interestingly, there was no change in the transcription level of *PTEN* (Fig. 3H–I). In a word, RNF126 changed the protein expression level by regulating the transcription level of the EGFR/PI3K/AKT signaling pathway, but RNF126 regulated PTEN without affecting its mRNA expression. Since RNF126 is an E3 ubiquitin ligase, we suspected that RNF126 might degrade PTEN through the ubiquitination to activate the EGFR/PI3K/AKT pathway.

### E3 ubiquitin ligase RNF126 interacts with PTEN

**Fig. 4:**
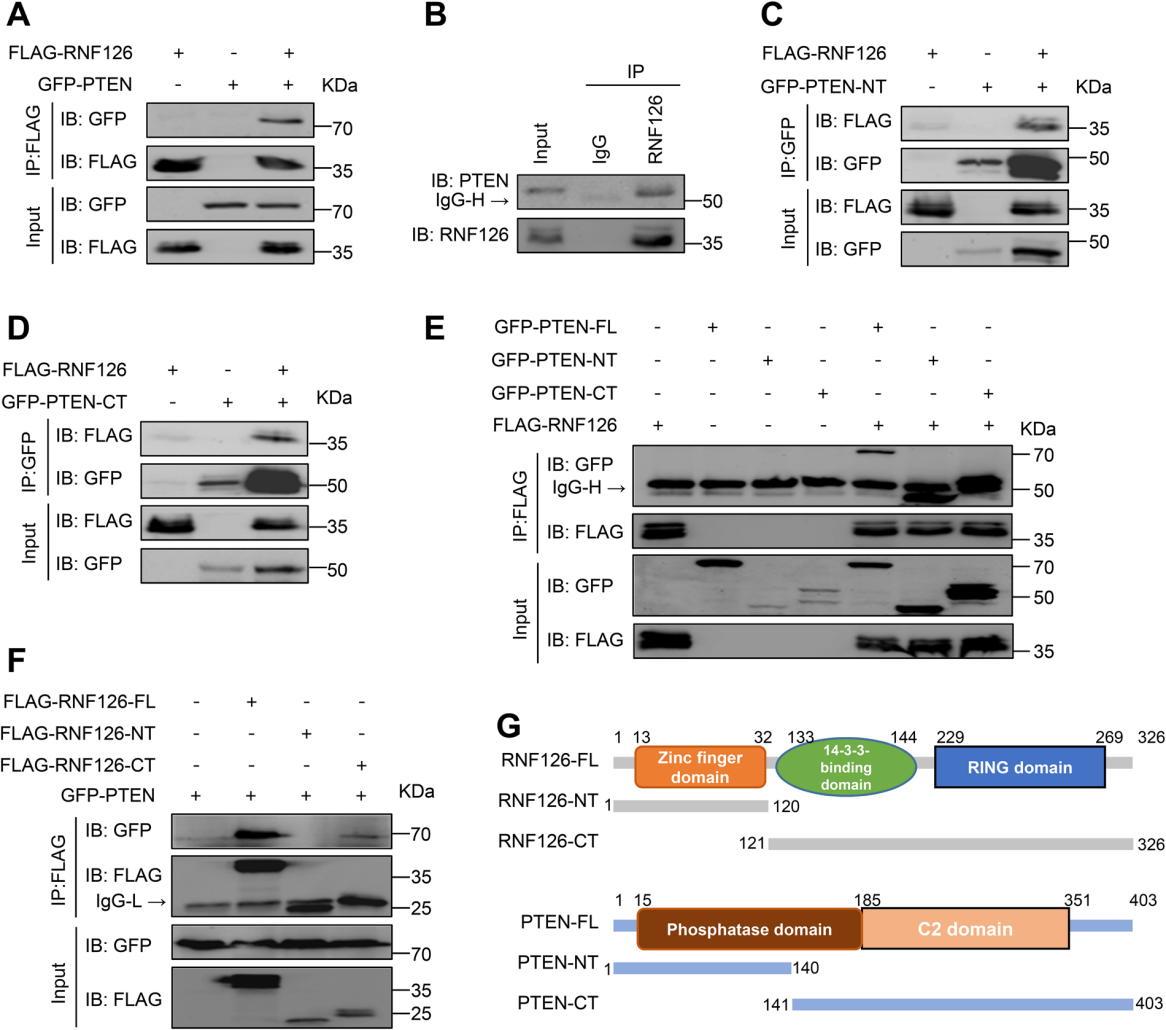
RNF126 interacts with PTEN. **A** The co-immunoprecipitation of GFP-PTEN and FLAG-RNF126 in 293T cells. The 293T cells were transfected with FLAG-RNF126 and GFP-PTEN plasmids for 36 h. **B** The endogenous co-immunoprecipitation of PTEN and RNF126 in T24 cells. **C–E** The interactions between full-length RNF126 and truncated forms of GFP-PTEN were observed by co-immunoprecipitation in 293T cells. **F** The interactions between full-length PTEN and truncated forms of FLAG-RNF126 were observed by co-immunoprecipitation in 293T cells. **G** Protein domains of RNF126 and PTEN. Numbers indicate how long the proteins were cut off. RNF126 protein was truncated between the RING finger domain and the Zn finger domain. PTEN protein was truncated to reserve the C2 domain.

To explore the mechanism of these changes in PTEN above, the co-immunoprecipitation analysis was performed to study the association between RNF126 and PTEN. When FLAG-RNF126 and GFP-PTEN were overexpressed in 293T cells, the interaction between FLAG-RNF126 and GFP-PTEN was detected (Fig. 4A). In addition, when endogenous RNF126 was immunoprecipitated by anti-RNF126 Ab, endogenous PTEN protein could be detected in T24 cells (Fig. 4B). To further investigate the exact domain of interaction between the two proteins, we constructed a series of truncation plasmids. It has been reported that the N-terminus has a Zn finger domain, the C-terminus has a RING finger domain and an AKT phosphorylation site in the middle in RNF126 protein^44^. So we separated the Zn finger domain and RING finger domain to construct the truncated forms of RNF126 (Fig. 4G). Co-immunoprecipitation analysis showed that both the N-terminus and C-terminus of the truncated forms of PTEN interacted with RNF126 (Fig. 4C–E). Specifically, only the C-terminus of RNF126 interacted with PTEN (Fig. 4F). Since RNF126 is an E3 ubiquitin ligase, we speculated that PTEN might be one of the substrates of RNF126.

### RNF126 regulates PTEN stability in an ubiquitin-proteasome-dependent manner

**Fig. 5:**
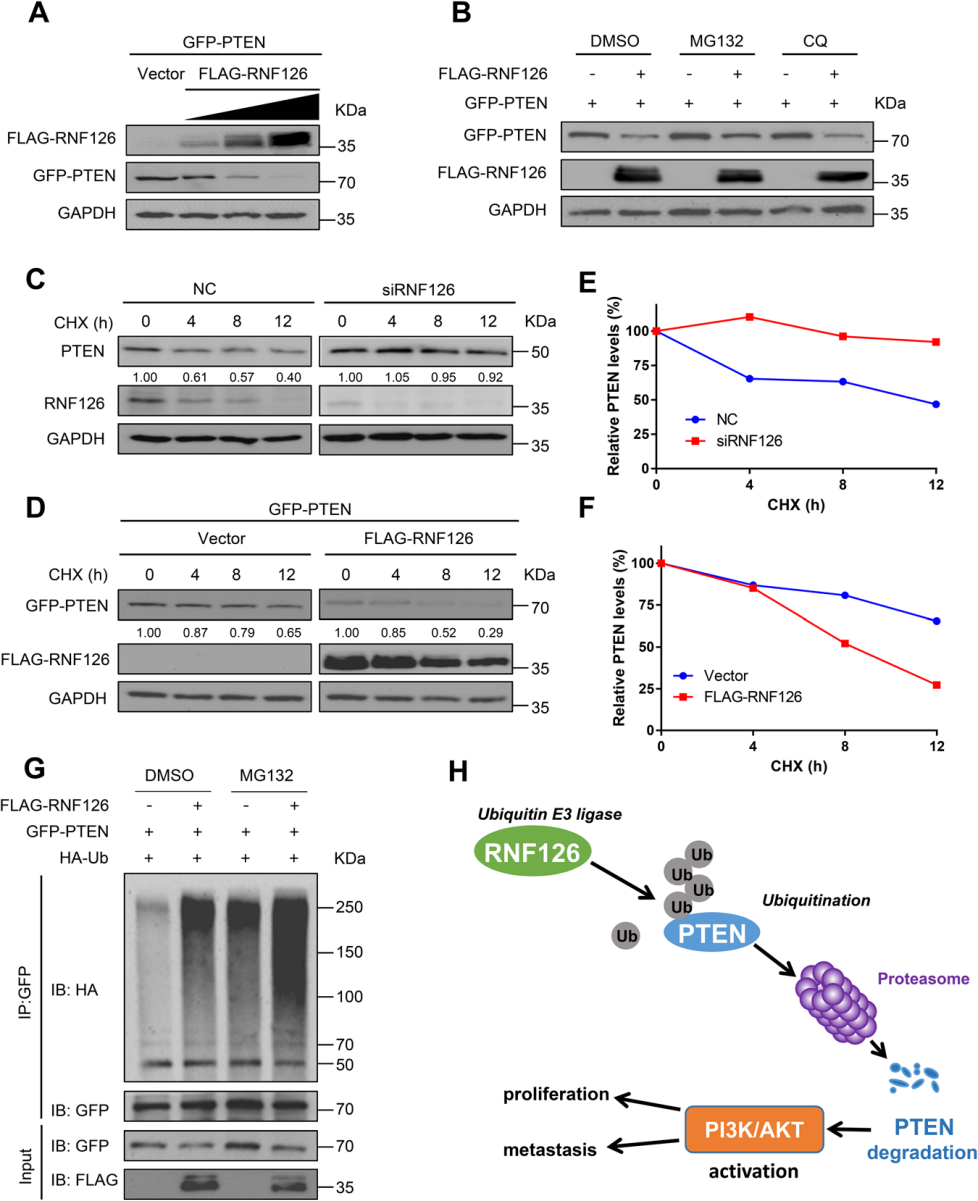
RNF126 regulates the stability of PTEN. **A** The 293T cells were transfected with GFP-PTEN and FLAG-RNF126 plasmids at different doses 36 h, then the cells were collected, and anti-GFP antibodies were detected by western blotting to determine the level of exogenous PTEN. **B** GFP-PTEN and FLAG-RNF126 were transfected into 293T cells for 24 h, then the cells were treated with dimethyl sulphoxide (DMSO), 10 μM MG132 (#S2619, Selleck) or 20 μM chloroquine (CQ, #S8808, Selleck) for 8 h. The cells were collected and anti-GFP antibodies were detected by western blot. **C** FLAG-RNF126 plasmid was transfected into 293T cells, and then 100 μg/ml cycloheximide (CHX, #S7418, Selleck) was respectively added at the specified time points for 0 h, 4 h, 8 h, and 12 h. Then, the cells were harvested, and western blot detected GFP antibodies to determine the half-life of GFP-PTEN protein. **D** The RNF126 siRNA was transfected into BCa 5637 cells for 36 h, and then 100 μg/ml CHX was added at specified time points for 0 h, 4 h, 8 h, 12 h, and collect cells. The half-life of endogenous PTEN protein was determined by western blot. **E**, **F** The ImageJ v1.45 software was used to quantified PTEN protein abundance. The relative level of PTEN protein plotted as indicated. **G** The in vivo ubiquitination assay showed that RNF126 poly-ubiquitinated PTEN. HA-Ub, GFP-PTEN and FLAG-RNF126 plasmids were transfected into 293T cells for 36 h. The cells were treated with 10 μM MG132 or DMSO for 8 h before harvest. **H** Graphic model of RNF126 affecting proliferation and metastasis through ubiquitination and degradation of PTEN in BCa.

To investigate whether RNF126 regulates the stability of PTEN, the 293T cells were transfected with a GFP-tagged PTEN plasmid and a FLAG-tagged RNF126 plasmid. We found that the protein level of exogenous PTEN decreased significantly as the expression level of exogenous RNF126 increased (Fig. 5A). Then, we found that RNF126-mediated degradation of PTEN was partly inhibited by the proteasome inhibitor MG132, but not the autophagy inhibitors chloroquine (CQ) (Fig. 5B). These results indicated that this process depended on the proteasome pathway. In addition, the cycloheximide (CHX) assay was performed to show that RNF126 deficiency led to a longer half-life of PTEN (Fig. 5C, E) and overexpression of RNF126 significantly accelerated the degradation of PTEN (Fig. 5D, F). More importantly, to test whether RNF126 was required for the ubiquitination of PTEN, in vivo ubiquitination assays were performed. Immunoprecipitation results indicated that overexpression of RNF126 significantly increased the poly-ubiquitination of PTEN in 293T cells and MG132 further enhanced the extent of poly-ubiquitination (Fig. 5G). Altogether, these data strongly suggested that RNF126 regulates the stability of PTEN through ubiquitination proteasome degradation manner (Fig. 5H).

### RNF126 promotes tumor proliferation and metastasis in a PTEN-dependent manner

**Fig. 6:**
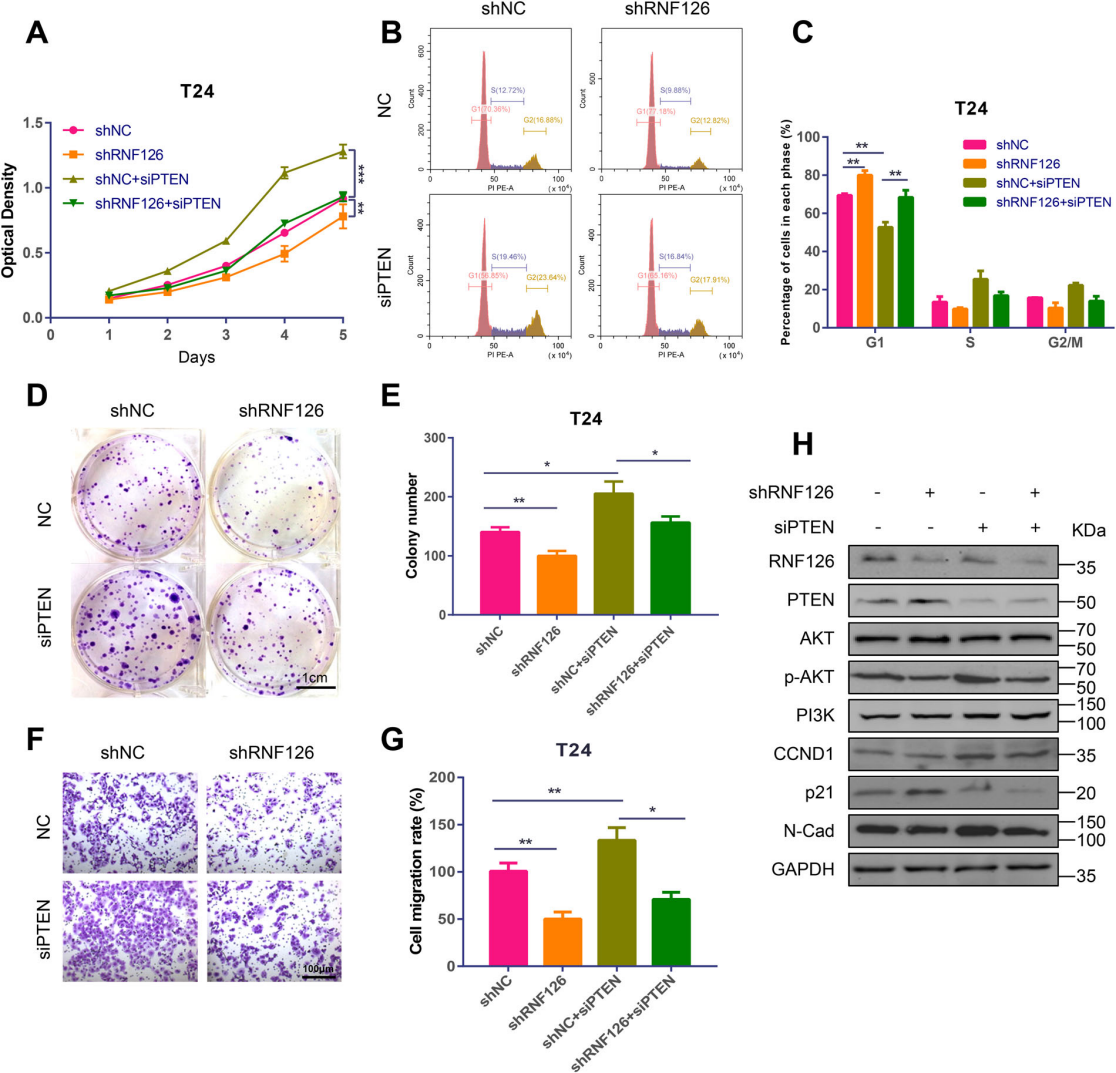
Depletion of RNF126 suppressed BCa cell proliferation and metastasis through upregulating PTEN. **A** The MTT assay evaluated the growth and viability of the T24 cell from day 1 until day 5. A lentiviral *shRNF126* and a lentiviral control group *shNC* stably transfected T24 cell were established. The cells were transfected with *siNC* (red line: *shNC* and orange line: *shRNF126*) or *siPTEN* (olive line: *shNC* + *siPTEN* and green line: *shRNF126* + *siPTEN*). **B**, **C** The flow cytometry analysis demonstrated the distribution of cells in different phases of cell cycle. The four groups were the same as above. **D** The colony formation assay showed the effect of *RNF126* knockdown on the cell survival of UMUC3 and T24 cells after the indicated transfection. Scale bar = 1 cm. **E** Colony numbers were counted and plotted as indicated. **F** The transwell assay evaluated cell migration of the *RNF126* or *PTEN* knockdown treated T24 cells. Scale bar = 100 µm. **G** The relative number of cell migration was statistically analyzed. **H** The western blotting indicated the protein level of RNF126, PTEN, PI3K, p-AKT, AKT, CCND1, p21, and N-Cad. Loading control was GAPDH. ***p < 0.001, **p < 0.01, *p < 0.05.

**Fig. 7:**
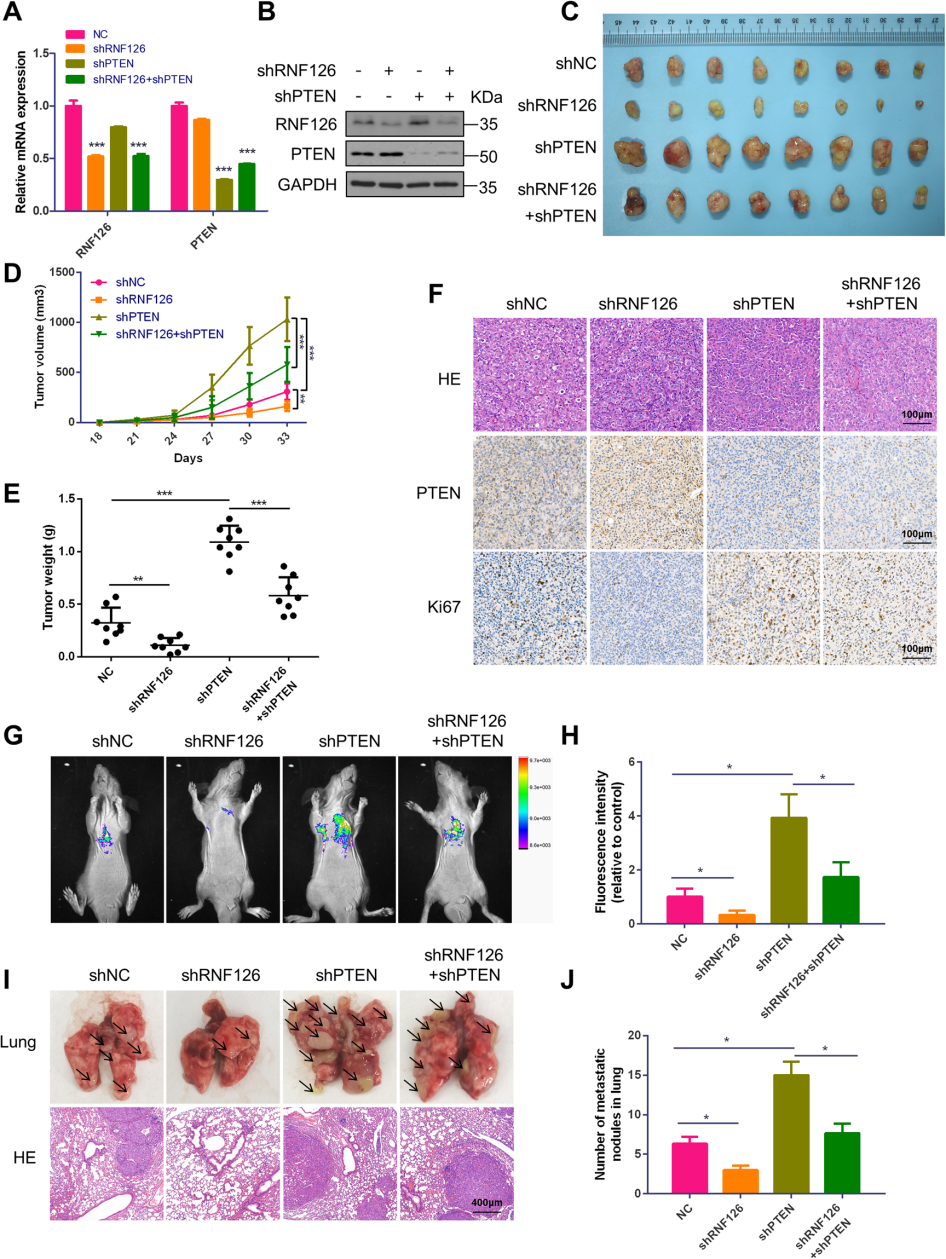
Effects of RNF126 and PTEN on BCa cell progression in vivo. **A**, **B** The qRT-PCR and western blotting showed the stable knockdown of *RNF126* and *PTEN* mRNA and protein levels in T24 cells. **C** *LV-control, LV-shRNF126*, *LV-shPTEN* and *LV-shRNF126* + *shPTEN* T24 cells were subcutaneously injected into four groups mice to generate the xenograft models. When the mice grew for 5 weeks, the xenografts were taken out and photographed (*n* = 8). **D** Tumor volume growth of BCa cells was measured twice a week until it grew for 5 weeks. **E** After the mice were sacrificed, the tumor weight was measured. **F** IHC demonstrated the expression of RNF126, PTEN and Ki67 proteins in tumors. Scale bar = 100 μm. **G**, **H** The mice’s tails were intravenous injected the *LV-control, LV-shRNF126*, *LV-shPTEN* and *LV-shRNF126* + *shPTEN* T24 cells to establish the pulmonary metastasis models (*n* = 3). The BCa cell migration capacity was demonstrated by the fluorescence of pulmonary metastases. **I** After injection for 8 weeks, the mice were sacrificed and the lungs were taken out. Then, the lungs were stained with HE. The arrows denote the tumors transferred to the lungs. Scale bar = 100 μm. **J** The quantification of pulmonary metastases was determined by counting the metastatic nodules. ***p < 0.001, **p < 0.01, *p < 0.05.

To clarify the specific role of PTEN and RNF126 in BCa and further determine whether RNF126-mediated tumor progression and metastasis require PTEN degradation, the *siPTEN* and the lentiviral *shRNF126* stable transfected T24 cells were prepared. Here, we used two lentiviral shRNA expression vectors to knockdown *RNF126* and the results showed that *shRNF126-1* and *shRNF126-2* had the same effects on cell cycle, proliferation and migration (Supplementary Fig. S4A–H). So we chose one of them for later experiments. MTT assay demonstrated that *shRNF126* greatly inhibited cell proliferation compared to the control group, and downregulation of *PTEN* promoted it (Fig. 6A). Flow cytometry results showed that deletion of *RNF126* increased the percentage of G1 phase cell release, while downregulation of *PTEN* decreased the percentage and restored the phenotype of *shRNF126* (Fig. 7B, C). The colony formation assay demonstrated that the colony number was decreased by *shRNF126* and rescued by *siPTEN* (Fig. 6D, E). Additionally, transwell migration experiments indicated that knocking down *PTEN* rescued the cell metastasis of BCa cells inhibited by *RNF126* deletion (Fig. 6F, G). Western blotting confirmed that RNF126 and PTEN were decreased in *shRNF126* and *siPTEN* group and demonstrated expression of several proteins, such as p-AKT, p21, N-Cad, changed by *shRNF126* and rescued by *siPTEN* (Fig. 6H). All in all, knockdown *RNF126* inhibits tumor cell phenotypes, in part by enhancing the protein stability of PTEN, therefore silencing *PTEN* on top of *shRNF126* rescues the inhibition in tumor phenotypes by *shRNF126*.

### Effects of RNF126 and PTEN on BCa growth and pulmonary metastasis in vivo

To identify the exact role of RNF126 and PTEN in vivo, *LV-shNC*, *LV-shRNF126*, *LV-shPTEN* and *LV-shRNF126* + *shPTEN* stable cell lines were established, the qRT-PCR and western blot analysis demonstrated the efficiency of *RNF126* and *PTEN* silencing by *shRNF126* and *shPTEN* (Fig. 7A, B). Subsequently, xenograft models were constructed (Fig. 7C), the *shRNF126* group grew slower and *PTEN* knockdown significantly promoted the growth. Furthermore, the tumor weight was significantly lower in the *shRNF126* group than in the negative control. However, *shPTEN* reversed the effect caused by *shRNF126* (Fig. 7D, E). The HE staining demonstrated the increased number of tumor cells in the *shPTEN* group, and the IHC staining indicated that PTEN expression was upregulated and Ki67 expression was downregulated in the *shRNF126* group compared with negative control (Fig. 7F). What’s more, *shRNF126* + *shPTEN* group restored all the results of *shRNF126* group.

In the same way, to study their BCa metastasis ability in vivo, the tails of nude mice were intravenously injected with the above four cell lines to established pulmonary metastasis models. After 8 weeks, we evaluated the metastasis ability by measuring the fluorescence of pulmonary metastases (Fig. 7G). As exhibited in Fig. 7G–J, *shRNF126* could significantly inhibit migration in vivo compared to the control group, and knockdown *PTEN* rescued its phenotype. The nodes of lung and HE staining displayed the pulmonary metastasis tumors (Fig. 7G, I). These results nicely fit the previous research about PTEN’s role as a tumor suppressor in BCa and revealed that RNF126 acts as an oncogene by suppressing PTEN’s functions.

## Discussion

Multiple E3 ubiquitin ligases play important roles in oncogenesis, cancer progression, prognosis, and chemo-resistance through the ubiquitin-proteasome pathway^45,46^. They have the potential to become novel therapeutic targets for human cancers. In the current study, we focused on the role of E3 ubiquitin ligases RNF126. Overexpression of *RNF126* in BCa tissues and cell lines was confirmed in the TCGA database and the qRT-PCR. We performed MTT assay, flow cytometry analysis, colony formation and transwell assay to validate the specific function of RNF126 in BCa. The results showed that the reduction of *RNF126* inhibited the proliferation and metastasis of BCa cells, arresting cells in the G1 phase, but had no obvious effect on apoptosis.

The previous studies showed that RNF126 promotes the repair of DNA double-strand breaks (DSBs) through NHEJ and HR^16,17^. Cisplatin has been widely used as the first-line drug of combination chemotherapies for patients with advanced BCa^47^. What’s more, cisplatin leads to the intracellular accumulation of DNA double-strand breaks (DSBs), responsible for cell apoptosis^48^. Our results indicated that *RNF126* depletion markedly increases the effect of cisplatin in inducing apoptosis in BCa cells. However, the specific mechanism of how *RNF126* depletion enhances cisplatin-induced DNA damage is unclear, and it remains to study deeply.

It has been well known that the PI3K/AKT pathway is regulated by receptor tyrosine kinases. Wang et al. reported that p-AKT and its key downstream genes decreased after silencing *RNF126* in tongue cancer cells^37^. Consistently, in this study, we found that *RNF126* depletion induces the downregulation of EGFR and AKT both in protein and mRNA levels. However, Smith et al. proved that RNF126 was shown to promote the ubiquitination and degradation of EGFR^9^. Although whether RNF126 regulates the PI3K/AKT pathway through this receptor still unknown. In a previous study, Lee et al. identified the activation of PI3K/AKT pathway induces PTEN ubiquitination, accelerating tumorigenesis in cervical cancer^49^.

Interestingly, our results showed that knockdown *RNF126* increased PTEN protein expression, but *PTEN* mRNA levels had no obvious change, suggesting a possible function of RNF126 in the PTM of PTEN. PTEN is a major tumor suppressor, which antagonizes PI3K/AKT signaling stimulated by growth factors by converting PIP3 to PIP2^27^. It seems to provide another way to explain the activation of the PI3K/AKT pathway by RNF126. As has been reported, the RING domain is indispensable for the E3 ligase activity, binding the substrate and the E2 ligase and transferring the ubiquitin from E2 ligase to the substrate^50,51^. The C-terminus containing RING domain of RNF126 interacts with PTEN, which strongly supports that RNF126 promotes the ubiquitination and degradation of PTEN through the proteasome pathway. The in vivo ubiquitination experiment confirmed our conjecture. The ubiquitination of PTEN regulates PTEN by promoting protein degradation and inhibition of phosphatase activity.

In vitro and in vivo investigations have revealed that PTEN acted as a tumor suppressor in BCa and PTEN silencing is tightly connected with the poor prognosis of BCa patients^52^. It has reported that microRNAs such as miR-103/107^53^, long non-coding RNAs such as lncRNA DUXAP8^54^, circular RNAs such as circ-ITCH^55^, anti-tumor drugs such as β-elemene^56^, and signaling pathways such as NF-κB^57^ are able to target PTEN in BCa cells, effecting the cell survival, proliferation, migration, invasion, apoptosis and chemoresistance of BCa. It seems to imply that the regulation of PTEN plays a significant role in the progression of BCa. Knockdown *RNF126* can reverse the growth and metastasis promoting effects of *PTEN* silencing, was that because silencing *PTEN* does not completely eliminate PTEN protein and so downregulating *RNF126* helps to boost the residual amounts of PTEN protein after silencing *PTEN*.

In the current study, for the first time, we found that RNF126 acts as a novel E3 ligase for PTEN through proteasome pathway ubiquitination. RNF126 plays a vital role in the progression of BCa and chemotherapy sensitivity to cisplatin treatment through the ubiquitination degradation of PTEN. Moreover, it is necessary to further elucidate the potential mechanism that affects RNF126 functions and carefully evaluate the influence of RNF126 in BCa. The ubiquitination sites on PTEN by RNF126 also need to be further investigated.

In summary, we revealed that RNF126 as E3 ubiquitin ligase promotes the ubiquitination and degradation of PTEN, thereby activating the PI3K/AKT signaling pathway to promote the tumorigenesis of BCa. Moreover, our findings provide evidence that RNF126 has the potential to become a new biomarker and possible therapeutic target in BCa.

## Materials and methods

### Cell culture and reagents

Human BCa cell lines BIU-87, T24, UMUC3, 5637, human bladder epithelial cells SV-HUC-1 and human embryonic kidney cells 293T were obtained from the Stem Cell Bank, Chinese Academy of Sciences in Shanghai. All cell lines were recently authenticated. The RPMI-1640 medium (Gibco, China) with 10% fetal bovine serum (FBS) was applied for T24, 5637, BIU-87 and SV-HUC-1 cells culturing. Dulbecco’s modified Eagle’s medium (Gibco, China) with 10% FBS was used for UMUC3 and 293T cells culturing.

Antibodies against RNF126 (ab183102, Abcam and sc-376005, Santa Cruz), PTEN (9559S, CST), GAPDH (sc-365062, Santa Cruz), E-Cad (3195S, CST), N-Cad (13116S, CST), CCND1 (2922, CST), CDK2 (ab32147, Abcam), CDK4 (12709, CST), p21(2947, CST), EGFR (4267, CST), PI3K (4257, CST), AKT (4685, CST), p-AKT (4060, CST), mTOR (ab32028, Abcam), p-mTOR (ab109268, Abcam), FLAG (F1804, Sigma), GFP (ab290, Abcam), HA (TA180128, OriGene), Ki67 (9449, CST), Ub (3933, CST) were purchased from indicated commercial sources.

### MTT assay

The single-cell suspension distinct BCa cells were prepared after transfection for two days, and seeded at a density of 4 × 10^3^ cells per well in 96-well plates. After incubation of 1–5 days, each well was added 20 μl of 5 mg/ml MTT (methyl thiazolyl tetrazolium, Sigma) solution. After incubation 4 h at 37 °C, every well was added 150 μl of DMSO for 10 min. Next, the plates were put in a microplate reader method (Category #SpectraMax M2, Molecular Equipment, USA) to measure the absorbance at 490 nm.

### Colony formation

After transfection for two days, 1 × 10^3^cells per well were plated in 6-well plates. After 10–14 days of culture, the supernatant was aspirated. Then, 4% paraformaldehyde (PFA) was used to fix cells for 30 minutes. Next, 0.1% crystal violet was used to crystallized cells. Statistical analysis was performed on the number of formed cell colonies.

### Transwell migration assay

The polycarbonate transwell filters (Corning, USA) was used to perform a transwell migration assay. Briefly, a cell suspension was prepared in serum-free medium after transfecting for two days, and 4 × 10^4^ cells per well were put in the transwell chamber. After incubating for 24 h at 37 °C, fix the cells in the lower chamber with 4% PFA, then stain with 0.1% crystal violet. Next, removed the cells in the upper chamber, and randomly count and photographed the cells left on another side.

### Wound healing assay

BCa cells were cultured to 80% in the six-well plate after transfection for two days. Then, the cells were scratched vertically with a small pipette tip, and washed with PBS to remove the scratched cells. Next, each well was added 200 μl culture medium and continue to grow for 24 h. Finally, the samples were taken to observe and photographed cell migration.

### Flow cytometry analysis

BCa cells were centrifuged and washed in cold PBS after transfecting for two days. For cell cycle analysis, the 1×DNA Staining Solution supplemented with propidium iodide (Multi sciences, China) was used to treat the cells. The cell apoptosis was assessed by the FITC Annexin V Apoptosis Detection Kit I (BD Biosciences, USA). Then the stained cells incubated for a half-hour at 25 °C in the dark. The flow cytometer (Cat. #FC500, Beckman, USA) was applied to analyze the distribution of cell cycle and apoptosis.

### Plasmids, siRNA and shRNA

We generated RNF126, RNF126-NT, RNF126-CT, PTEN, PTEN-NT, and PTEN-CT plasmids according to the ClonExpress^®^ II One Step Cloning Kit (Vazyme Biotech Co., Ltd). The primers of all plasmid constructions were listed in Supplementary Table S1. The cDNA of RNF126, RNF126-NT, and RNF126-CT were cloned into a 3×FLAG-pcDNA3.1 vector. The cDNA of PTEN, PTEN-NT, and PTEN-CT were cloned into a GFP-pcDNA3.1 vector. The DNA constructs sequences were systematically verified by DNA sequencing.

The *RNF126*-si1: 5’-GCCGGAUUAUAUCUGUCCATT-3’, *RNF126*-si2: 5’-GCAUCUUCGAUGACAGCUUTT-3’, *RNF126*-si3: 5’-CCAACGGCCUGGAUGCCAUTT-3’, siPTEN: 5’-ATCGATAGCATTTGCAGTATA-3’ and siNC: 5’-UUCUCCGAACGUGUCACGUTT-3’ were purchased from GenePharma Co., Ltd, Shanghai, China. The siRNAs and plasmids were transfected into cells with Opti-MEM culture medium, Lipofectamine^®^ 3000 and P3000^TM^ (Invitrogen).

The lentiviral shRNA expression vectors for human RNF126, human PTEN and control were also purchased from GenePharma Co., Ltd. The *shRNF126*-1: 5’-CCAACGGCCTGGATGCCAT-3’, *shRNF126*-2: 5’-GCCTCACGGGACAGAACACTT-3’, *shPTEN*: 5’-ATCGATAGCATTTGCAGTATA-3’ and shNC: 5’-TTCTCCGAACGTGTCACGT-3’ were also purchased from GenePharma Co., Ltd. The lentiviral shRNA expression vectors were transfected into cells with 6 μg/ml polybrene. After transfection for two days, the infected cells were selected by treatment with 2 μg/ml puromycin.

### Total RNA isolation and qRT-PCR

We used RNeasy Mini Kit (Cat. #74101, Qiagen, Germany) to isolate total RNA of BCa cells according to the protocol. Then, the reverse transcription reaction was conducted to synthesize cDNA according to ReverTrace qRT-PCR Kit (Toyobo, China). Each reaction of qRT-PCR was performed with 3 μl of primers, 4.5 μl of cDNA, and 7.5 μl iQTM SYBR^®^ Green Supermix (Bio-Rad, USA). The primer sequences for qRT-PCR were listed in Supplementary Table S2. The cycle threshold (Ct) was calculated compared to GAPDH.

### Total protein isolation and western blotting

The cells were centrifuged and lysed on ice for a half-hour with RIPA buffer supplemented with protease inhibitor and phosphatase inhibitor (Sigma-Aldrich, USA). Then, the cells were treated by an ultrasonic crusher for 10 s and centrifuged at 14,000 × *g* for 10 min. Protein separation and detection were performed as previously described^58^. Finally, immune response bands were exposed in the electromagnetic interference XRS imaging system (Bio-Rad, USA).

### Co-immunoprecipitation

Cells were collected after transfection for 48 h. Then, a cell lysis buffer containing a proteasome inhibitor was added, lysed on ice for a half-hour, and centrifuged at 14,000 × *g* at 4 °C for 10 min. A small amount of supernatant was used as Input for western blot analysis. The magnetic beads bound to the corresponding antibodies were added to the remaining supernatant and incubated at 4 °C with slow shaking overnight. The antigen-antibody magnetic beads were conjugated. After the immunoprecipitation reaction, the magnetic bead coupling complex was washed with the buffer. Next, add 15 μl of 1 × SDS buffer and cook at 100 °C for 5 min. The proteins were separated by SDS-PAGE electrophoresis. Finally, western blotting was performed to identify the interacting proteins.

### Xenograft model and pulmonary metastasis model

The 4-weeks old male BALB/c-nu mice, specific pathogen free (SPF), were purchased from Beijing Vital River Laboratory Animal Technology Co., Ltd. and fed in the SPF environment of laboratory animal facility of Zhongnan Hospital. After adaption for 5–7 days, the mice were randomly divided into four groups and bilateral subcutaneously inoculated with BCa T24 *LV-control, LV-shRNF126*, *LV-shPTEN* or *LV-shRNF126* + *shPTEN* cells (5 × 10^6^ cells/100 µl) to established the xenograft model (*n* = 4). In addition, the mice tail intravenous injecting the above four cell lines (1 × 10^6^ cells/100 µl) to established pulmonary metastasis models (*n* = 3). The tumor length (a) and width (b) were measured twice a week. The tumor size = a × b^2^/2 mm^3^ ^59^. The xenograft growth was monitored for 5 weeks. Then, the mice were sacrificed to get fresh tumors and the tumor weight was measured. After growth for 8 weeks, the fluorescence intensity of tumors with GFP was quantified by the Living Image software (Caliper Life Sciences). We were blinded to the group allocation when assessing the animal model. The tumors and lungs were then fixed in 4% PFA. Next, the xenografts and pulmonary metastases were analyzed by hematoxylin and eosin (HE) staining and immunohistochemical (IHC) staining for the indicated antibodies. All animal experiments were approved by the Wuhan University Institutional Animal Care and Use Committee.

### Immunohistochemistry

PTEN and Ki-67 protein expressions in BCa xenografts were identified by IHC. First, fresh tumors were fixed in 4% PFA for 24 h. Then, they were embedded in paraffin, and cut into 5 μm sections. Next, the sections were IHC stained by the PTEN antibody (1:200) and Ki67 antibody (1:200). The DAB chromogen was used to incubated and then hematoxylin was used to counterstain. Finally, the sections were analyzed under a light microscope.

### Statistical analyses

Data were expressed as mean ± SD from three individual experiments. The statistical differences were analyzed by two-tailed student’s *t*-tests. The GraphPad Prism 7.00 was used for all statistical analysis. Images were analyzed and quantified using the ImageJ v1.45 software. We considered *p* < 0.05 has statistically significant.

## Supplementary information

Supplementary Tables

Supplementary figure legends

Supplementary Figure S1

Supplementary Figure S2

Supplementary Figure S3

Supplementary Figure S4
