# Supplementary material for: E3 ubiquitin ligase RNF126 affects bladder cancer progression through regulation of PTEN stability
Source: Cell Death Dis. 2021 Mar 4;12(3):239. doi: 10.1038/s41419-021-03521-1 (PMC7933351; doi:10.1038/s41419-021-03521-1)
Supplement: Supplementary file 1 — Supplementary Tables [file 41419_2021_3521_MOESM1_ESM.docx]

**Supplementary Tables**

**Supplementary Table S1. List of primers for plasmid construction.**

| Gene | Forward primer | Reverse prime |
| --- | --- | --- |
| RNF126 | GATAAAGGTCACCCAGGATCCATGGCCGAGGCGTCGCCG | TGATGGATATCTGCAGAATTCTCACGAGTTGCTTGTGGCG |
| RNF126-NT | AAAGGATCCATGGCCGAGGCGTCG | AAAGAATTCTCAGTGCCGGGACGG |
| RNF126-CT | AAAGGATCCATGCGGTACGGCGCC | AAAGAATTCTCACGAGTTGCTTGT |
| PTEN-NT | AAAAAGCTTATGACAGCCATCATC | AAAGGATCCGGTCCTTACTTCCCC |
| PTEN-CT | AAAAAGCTTATGAGAGACAAAAAG | AAAGGATCCGACTTTTGTAATTTG |

**Supplementary Table S2. List of primers for RT-qPCR.**

| Gene | Forward primer | Reverse prime |
| --- | --- | --- |
| RNF126 | CTCAAACCCTATGGACTACGC | GGCCTGTGTTTTCAAACTGAT |
| EGFR | AGGCACGAGTAACAAGCTCAC | ATGAGGACATAACCAGCCACC |
| AKT | GTCATCGAACGCACCTTCCAT | AGCTTCAGGTACTCAAACTCGT |
| PI3K | CCACGACCATCATCAGGTGAA | CCTCACGGAGGCATTCTAAAGT |
| CCND1 | GCTGCGAAGTGGAAACCATC | CCTCCTTCTGCACACATTTGAA |
| PTEN | TGGATTCGACTTAGACTTGACCT | GGTGGGTTATGGTCTTCAAAAGG |
| GAPDH | GGAGCGAGATCCCTCCAAAAT | GGCTGTTGTCATACTTCTCATGG |
