# Supplementary material for: E3 ubiquitin ligase RNF126 affects bladder cancer progression through regulation of PTEN stability
Source: Cell Death Dis. 2021 Mar 4;12(3):239. doi: 10.1038/s41419-021-03521-1 (PMC7933351; doi:10.1038/s41419-021-03521-1)
Supplement: Supplementary file 2 — Supplementary figure legends [file 41419_2021_3521_MOESM2_ESM.docx]

**Supplementary Figure legends**

**Supplementary Figure S1. Expression of RNF126 and RNF126 promoter methylation in BCa of TCGA samples.** **(A-B)** Expression of RNF126 in BLCA based on the patient’s gender and age. **(C)** Expression of RNF126 in BLCA based on TP53 mutation status. **(D-E)** Expression of RNF126 in BLCA based on nodal metastasis status and individual cancer stage. **(F-L)** RNF126 promoter methylation profile based on different influencing factors indicated in each graph.

**Supplementary Figure S2. Overexpression of RNF126 promotes BCa cell proliferation and migration. (A)** Western blotting of FLAG-RNF126 showed the overexpression of RNF126. **(B-C)** MTT assay of overexpression of RNF126 in T24 and UMUC3 BCa cells. **(D)** The flow cytometry analysis showed the percentage (%) of cells in different phases of the cell cycle. Vector and FLAG-RNF126 were transfected into T24 cells for two days. **(E)** The transwell assay evaluated cell migration of the Vector and FLAG-RNF126 treated BCa cells. Scale bar = 100 µm. **(F)** The relative number of cell migration were statistically analyzed. ***p < 0.001, **p < 0.01, *p < 0.05.

**Supplementary Figure S3. RNF126 potentiates the chemoresistance in BCa.** **(A)** Apoptosis of UMUC3 cells was demonstrated by flow cytometry with 1 μM, 2 μM, 3 μM, 5 μM of cisplatin treatment or DMF for 24 h. **(B-C)** Apoptosis of negative control and siRNF126-transfected BCa cells was demonstrated by flow cytometry with 5 μM of cisplatin (#S1166, Selleck) treatment or N, N-Dimethylformamide (DMF, #S6192, Selleck) for 24 h. **(D-E)** The apoptotic rates (%) of T24 and UMUC3 cells were statistically analyzed. The cisplatin treatment and siRNA were indicated. ***p < 0.001, **p < 0.01, *p < 0.05.

**Supplementary Figure S4. RNF126 deficiency affects the cell cycle and inhibits BCa cell proliferation and migration. (A)** The MTT assay evaluated the growth and viability of the T24 cell from day 1 until day 5. Two lentiviral *shRNF126*-1*, shRNF126*-2 and a lentiviral control group *shNC* stably transfected BCa T24 cells were established. **(B)** The western blotting indicated the protein level of RNF126, N-Cad, CDK2, CDK4, CCND1, EGFR, PI3K, p-AKT, AKT and PTEN. The loading control was GAPDH. **(C-D)** The flow cytometry analysis demonstrated the distribution of cells in different phases of the cell cycle. **(E)** The colony formation assay showed the effect of RNF126 knockdown on the cell survival T24 cells. Scale bar = 1 cm **(F)** Colony numbers were counted and plotted as indicated. **(G)** The transwell assay evaluated cell migration of the RNF126 knockdown treated T24 cells. Scale bar = 100 µm. **(H)** The relative number of cell migration was statistically analyzed. ***p < 0.001, **p < 0.01, *p < 0.05.
