# Supplementary figures and images for: E3 ubiquitin ligase RNF126 affects bladder cancer progression through regulation of PTEN stability
Source: Cell Death Dis. 2021 Mar 4;12(3):239. doi: 10.1038/s41419-021-03521-1 (PMC7933351; doi:10.1038/s41419-021-03521-1)

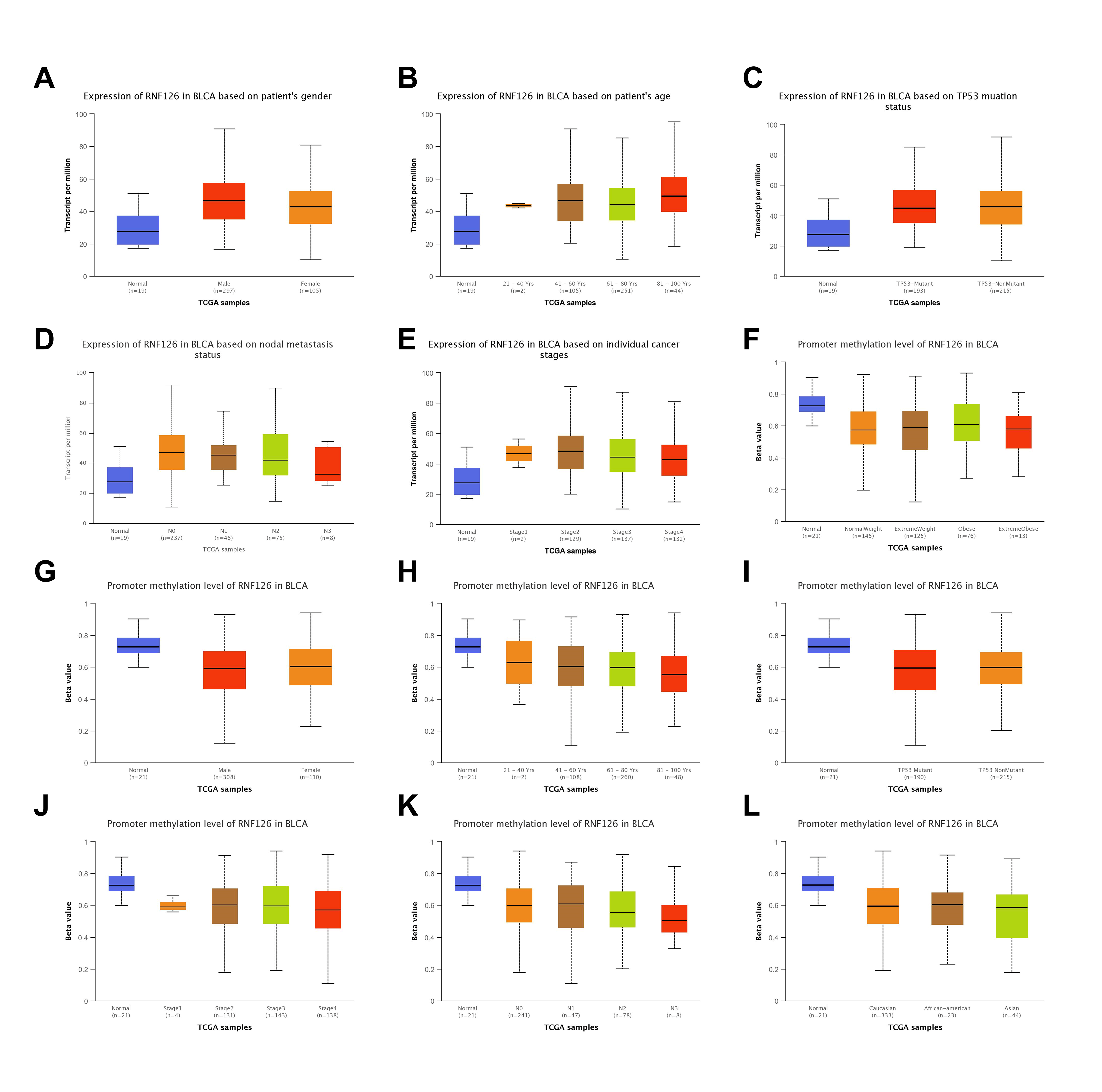

Supplement: Supplementary file 3 — Supplementary Figure S1 [file 41419_2021_3521_MOESM3_ESM.tif]

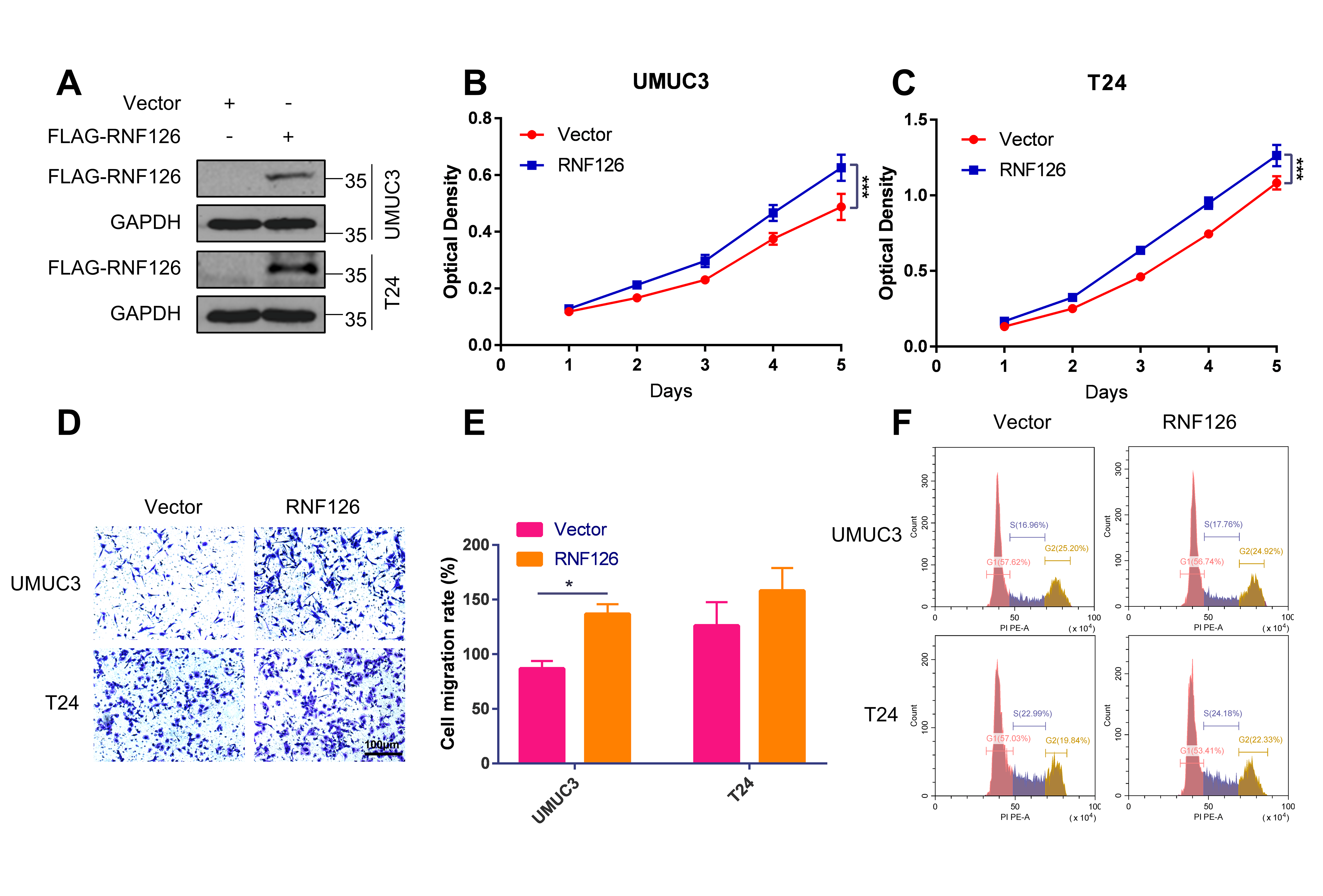

Supplement: Supplementary file 4 — Supplementary Figure S2 [file 41419_2021_3521_MOESM4_ESM.tif]

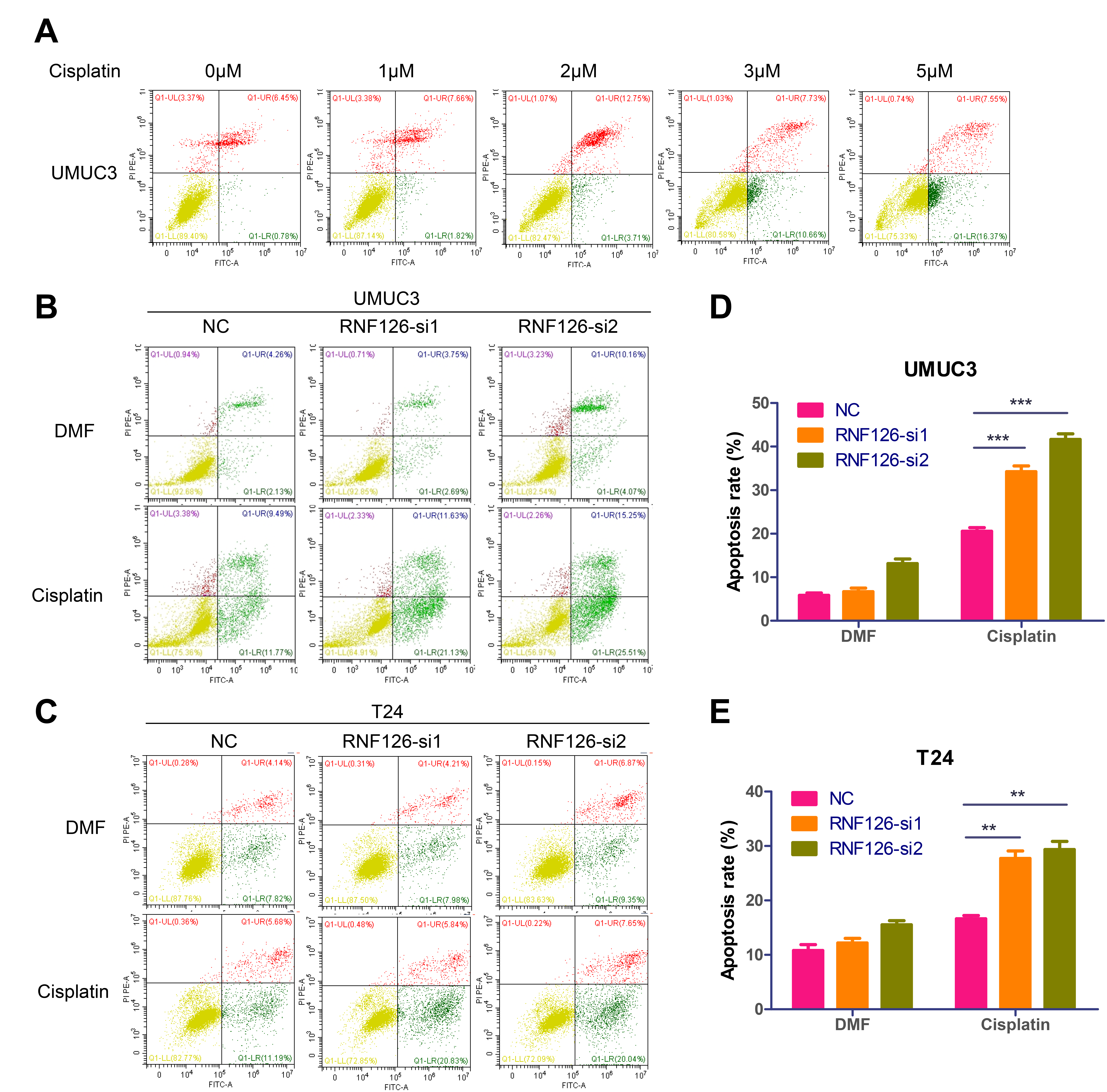

Supplement: Supplementary file 5 — Supplementary Figure S3 [file 41419_2021_3521_MOESM5_ESM.tif]

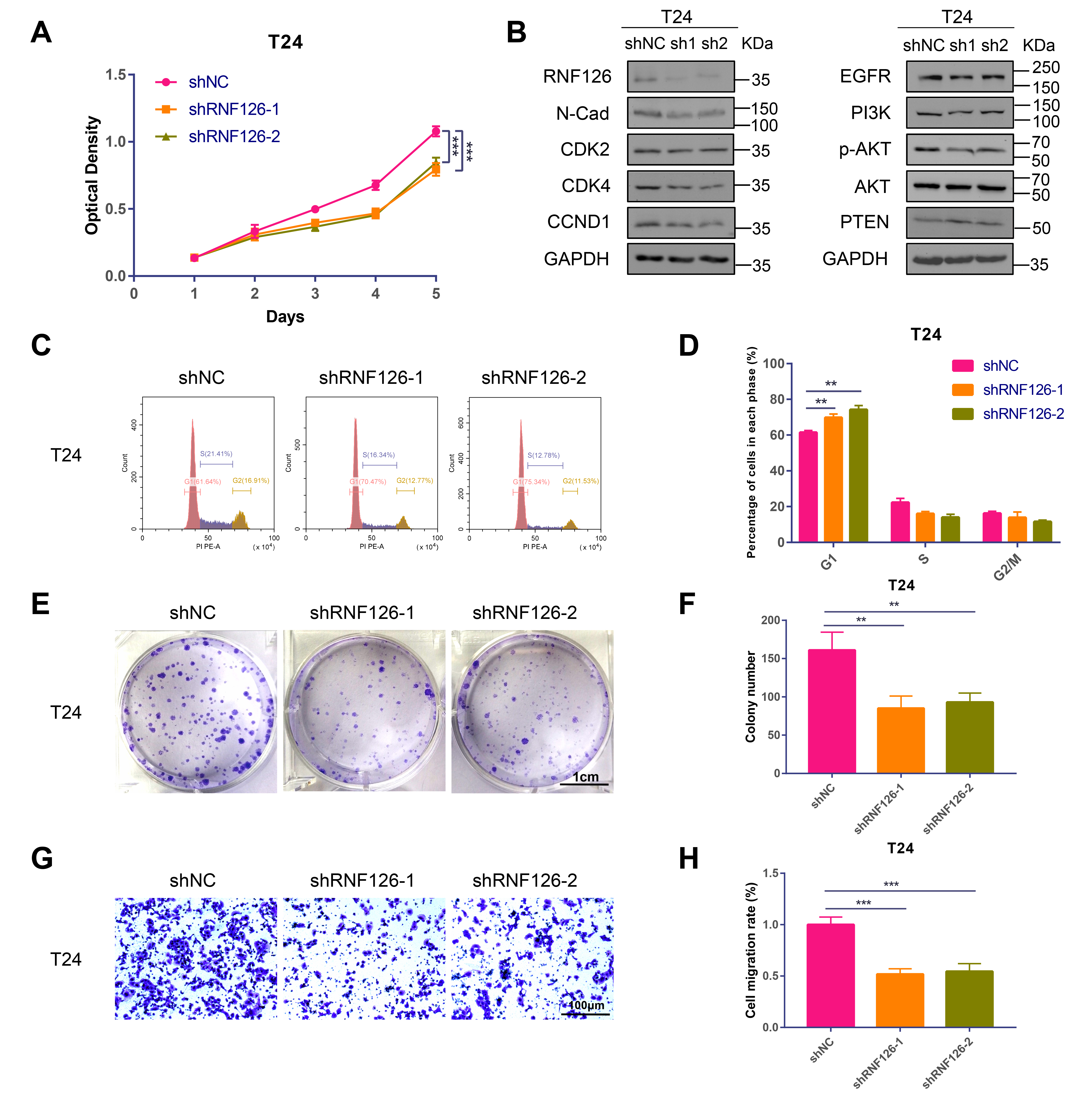

Supplement: Supplementary file 6 — Supplementary Figure S4 [file 41419_2021_3521_MOESM6_ESM.tif]
